# Cellular Inheritance

**DOI:** 10.1371/journal.pbio.0040446

**Published:** 2006-12-05

**Authors:** Emma Hill

Biological continuity relies on successful cell division. Research over the years has provided a good understanding of how cells divide to form two daughter cells through mitosis. During mitosis, chromosomes are duplicated and divied up between the cells to provide each daughter cell with a complete copy of the organism’s genome. The cell, however, doesn’t contain only genomic DNA but can accumulate damage in the form of misfolded proteins. How does the cell discard this unwanted material during mitosis?

Many diseases, including Huntington and Parkinson disease, can be attributed to protein misfolding that aggregates and accumulates in the cell. The underlying genes have nucleotide triplet-repeat mutations, which produce a protein with an expanded run of the same amino acid, commonly glutamine. Proteins with such polyglutamine stretches fold and function incorrectly. Misfolded proteins are generally targeted for degradation by the cell. However, at some point, the cellular mechanisms are overwhelmed, and aggregated protein will accumulate within the cell as aggresomes.

What happens next to these cells in terms of cell division was the question that Maria Rujano, Harm Kampinga, and colleagues set out to investigate. Can cells with accumulated damage undergo cell division and complete mitosis? And if so, what happens to the aggresome? These researchers found intriguing evidence of a system in eukaryotic cells (which contain nuclei and other double-membraned organelles) that distributes damage asymmetrically, with one daughter cell inheriting the aggresome and the other being damage-free. In cases of polarized cell division (where one cell becomes committed to a specific fate and the other doesn’t), this asymmetric mitosis favors leaving the long-lived committed daughter cell damage-free.

The researchers investigated multiple eukaryotic cell systems starting with human and hamster cells. They engineered the cells to transiently express a modified version of the *huntingtin* gene with a glutamine repeat that causes misfolding. As expected, a large number of cells had aggresomes, which allowed the authors to investigate whether the cells could undergo mitosis and divide and then determine what happened to the aggresomes. Cells with severe levels of damage were unable to progress through mitosis. However, in the single-aggresome–containing cells, the cell appeared normal throughout all phases of mitosis. In addition, only one daughter cell inherited the damage. Time-lapse imaging confirmed these results and also found that cells with aggresomes do take a little longer to complete mitosis than normal cells. So it seems that cells with aggresomes that are formed from expanded polyglutamine repeats are able to successfully complete mitosis.

To take this observation a step further, the authors looked to see what happens in the dividing cells of polarized tissues. For this they make use of two systems: intestinal crypt cells from two human patients with the neurodegenerative disorder spincerebellar ataxia type 3 and *Drosophila* neuroblast stem cells expressing a mutated polyglutamine form of the *huntingtin* gene. Because both of these cells divide to produce one short-lived daughter cell and one long-lived differentiated cell, the authors could investigate how accumulated damage was distributed between the two different daughter cells.

**Figure:**
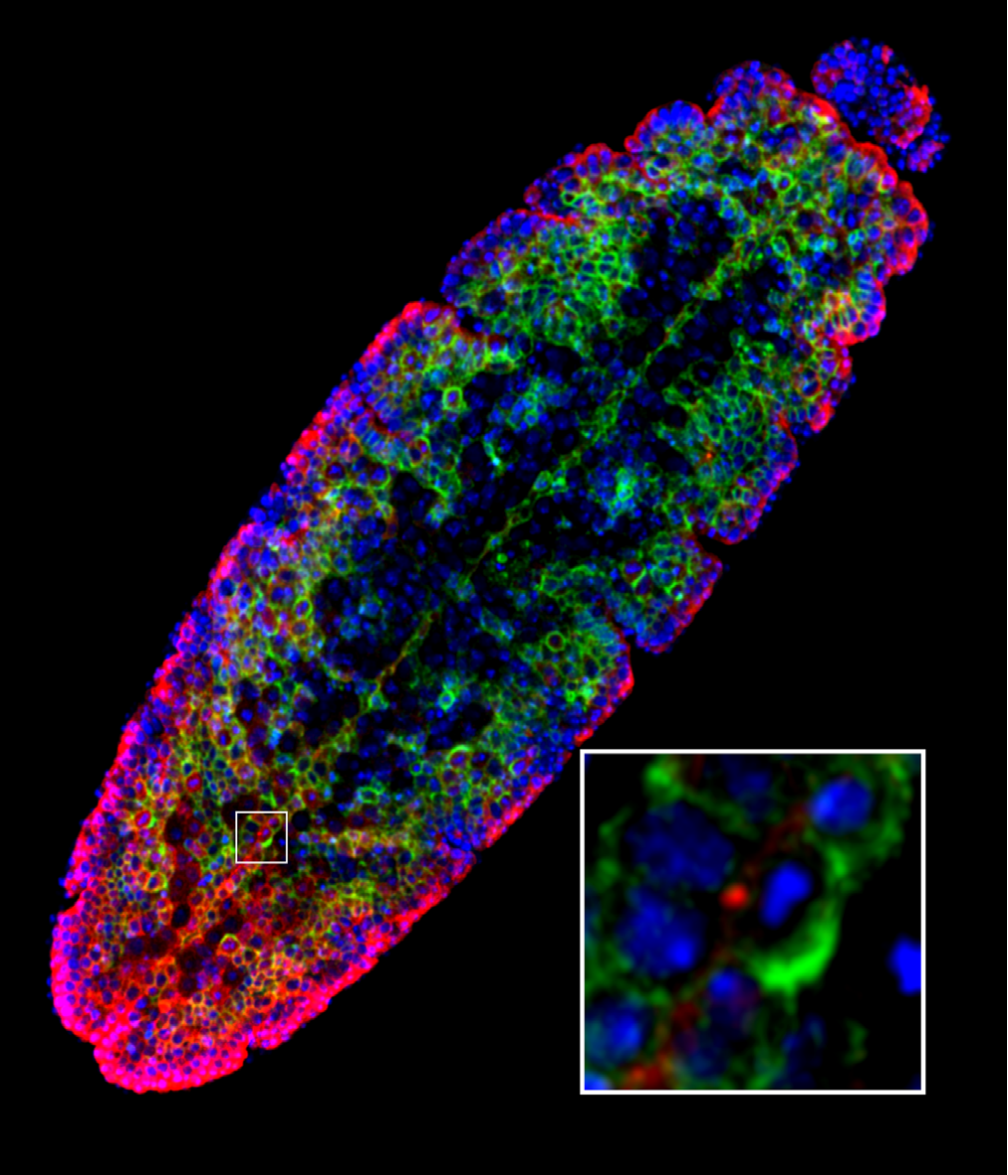
Aggregates of disease-associated misfolded or stress-damaged proteins can be stored at the microtubule organizing center and are inherited during mitosis with a polarity that ensures preservation of the long-lived progeny.

In the human system in which the stem cells give rise to one short-lived committed progenitor and differentiated cells, the authors saw that the stem cells themselves, which should in theory have accumulated aggregates over their longer lives, never actually contain aggresomes, whereas the committed and differentiated cells from these samples do contain damaged inclusion bodies. This is consistent with asymmetric inheritance of aggresomes by the shorter-lived non–stem cell after division. At this time, however, the researchers are unable to verify this hypothesis, because no mitotic stem cells are detected in this model.

In the *Drosophila* model, the neuroblast stem cells divide into one neuroblast (that will undergo several rounds of division before succumbing to a natural death at the end of embryogenesis) and one fate-committed ganglion mother cell (GMC) (that will go on to become a long-lived glial cell). By studying *Drosophila* embryos, the authors could visualize both expression of the mutated *huntingtin* gene and aggresome formation. They identified mitotic neuroblast cells, all of which expressed the mutated form of *huntingtin*, though few contained aggresomes. More interestingly, in all of the mitoses analyzed, the aggresome-like inclusion was inherited by the neuroblast daughter cell resulting in formation of a damage-free GMC. These observations provide strong evidence that these neural precursor cells undergo asymmetric distribution of aggregated proteins with a polarity, such that the long-lived committed daughter cell is favored and does not inherit the damage.

So it seems that damage-riddled cells can still divide and complete mitosis. Rujano and colleagues show this to be true in several different systems. Indeed, cells appear to have developed a clever damage-limitation system to ensure that specific long-lived daughter cells are not encumbered with damage from the parent cell. Future research will hopefully shed light on how this decision is made and what the mechanisms underlying this system are.

